# Injections of Osmium Acid in Neuralgia

**Published:** 1886-06

**Authors:** 


### Injections of Osmium Acid in Neuralgia.

In a meeting of the Petersburg Medical Society, Dr. B. M. Schapiro recently delivered a lecture on the treatment of neuralgia by subcutaneous injections of osmium acid (*St. Petersburg Med. Weekly Journal*, 26, 27, 1885.) He first mentioned the observation of Frankel, that the acid exerts a disturbing influence on the substance of the nerves and muscles, and greatly diminishes their activity. In nerves an epineuritis develops, which compresses the fibres, while in muscles an interstitial inflammation soon leads to atrophy of the muscular tissue. Living nerve fibres assume under the influence of the drug a black tint, while the neighboring tissue becomes denser, and motor nerves situated near occasionally suffer decidedly in consequence of these injections, a fact to be well noted.

Dr. Schapiro studied the effect of the acid in eight cases of neuralgia of the fifth nerve. The pains attacked all branches of the nerve, and all cases were obstinate and of long duration. The result was a complete cure in five cases (62.5 per cent.), in two cases a decided improvement, and in one case no effect whatever. The two greatly improved cases were of the most violent and obstinate nature, and the want of success of the most severe therapeutical procedures warranted the belief that the nerves had undergone an incurable morbid change at their origin.

The following solution was employed by Schapiro for the purposes of the hypodermic medication:

| | | |
|---|---|---|
| R. | Acidi osmici, | gr. 1⅔. |
| | Aquae destill., | f℥jss. |
| | Glycerin. pur., | f℥j. |
| M. | In vitro nigro. | |

This solution, according to Schapiro, can be kept in a good condition from two to three weeks, a fact of importance if one takes into consideration the high price of the drug, while the simple watery solution is decomposed within a day or two. The number of injections in each individual case varied from one to twelve; in the beginning five drops, and later a slightly increased quantity were employed. In every case where the injection was made into the tissues directly over the seat of the pain perfect analgesia developed, but the pain occasionally then attacked neighboring branches of the same nerve, which before had not been painful. Other deleterious effects were never observed by Schapiro, only one lady, highly nervous and subject to epileptic fits, fainted immediately after the injection.

In cases where all other therapeutical procedures seem to be without effect, or where the intensity of the pain necessitates immediate relief, the hypodermic application of osmium acid may, therefore, well be worth a trial.

